# Tonic Cold Pain Temporal Summation and Translesional Cold Pressor Test-Induced Pronociception in Spinal Cord Injury: Association with Spontaneous and Below-Level Neuropathic Pain

**DOI:** 10.3390/healthcare12222300

**Published:** 2024-11-17

**Authors:** Marta Ríos-León, Elena Demertzis, Ramiro Palazón-García, Julian Taylor

**Affiliations:** 1 Sensorimotor Function Group, Hospital Nacional de Parapléjicos (SESCAM), 45071 Toledo, Spain; elena.demertzis@aulss6.veneto.it (E.D.); ramiropalazon@yahoo.es (R.P.-G.); juliantaylorgreen2@gmail.com (J.T.); 2 Instituto de Investigación Sanitaria de Castilla-La Mancha (IDISCAM), 45071 Toledo, Spain; 3 Rehabilitation Unit, High Specialization Rehabilitation Hospital of Motta di Livenza, Motta di Livenza, 31045 Treviso, Italy; 4 Rehabilitation Department, Hospital Nacional de Parapléjicos (SESCAM), 45071 Toledo, Spain; 5 Harris Manchester College, University of Oxford, Oxford OX1 3TD, UK

**Keywords:** conditioned pain modulation, temporal summation of pain, pressure pain threshold, spinal cord injuries, neuropathic pain

## Abstract

**Background/Objectives**: Although increased nociceptive excitability and deficient endogenous pain modulation are considered key features of pronociception and central sensitization, their contribution to neuropathic pain (NP) characteristics in SCI is unclear. The aim of this study was to characterize tonic cold perception and endogenous pain modulation in individuals with and without SCI-NP, considering the stage and severity of SCI and, secondarily, NP phenotype. **Methods**: Temporal summation of pain (TSP) and neuropathic features were assessed using the numerical rating scale (NRS) and Douleur Neuropathique 4 screening questionnaire (DN4) during the tonic cold pressor test (CPT, 12 °C 60 s) applied to the dominant hand and foot. CPT-induced pronociception was assessed as change in algometer pressure pain thresholds (PPTs) measured at the V2, C6, and L4 dermatomes. **Results**: A total of 72 individuals were recruited (age-sex-matched noninjured, n = 24; SCI-NP, n = 24; SCI-noNP, n = 24 [AIS A: n = 12, AIS B-D: n = 12; subacute SCI: n = 12, chronic SCI: n = 12]). TSP in response to the foot CPT was higher in subacute compared to chronic incomplete SCI-NP, while TSP to the hand CPT was significantly higher in chronic compared to the subacute complete SCI-NP group. Evoked pain intensity during the hand CPT correlated with duration of below-level SCI-NP. The hand CPT induced widespread pronociception (lower PPT), which correlated with 7-day non-evoked (spontaneous) pain intensity in individuals with incomplete SCI-NP. Individuals with below-level NP, but not at-level NP, showed higher TSP during the foot CPT and greater hand CPT-induced L4 dermatome pronociception. **Conclusions**: Collectively, measurements of above and below-level temporal summation of pain and translesional-induced pronociception in the SCI-NP group highlight the role of these mechanisms in widespread central sensitization, spontaneous pain intensity, and spinothalamic tract hyperexcitability, especially in individuals diagnosed with below-level NP.

## 1. Introduction

Spinal cord injury (SCI) is a highly disabling and devastating pathology with increasing prevalence globally [1]. In fact, neuropathic pain (NP) affects over 50% of individuals with SCI, interfering with daily life and rehabilitation [2,3,4]. SCI-NP has been defined as at-level pain within three dermatomes below the neurological level of injury, and below-level pain in more distal dermatomes [5]. The management of NP is challenging and unsatisfactory [4,6], due to the multiple pathophysiological mechanisms throughout the neuroaxis that are not fully understood [4,7]. SCI-NP has been associated with lesions of the spinothalamic tract (STT) and neuronal hyperexcitability, corroborating clinical signs of spontaneous pain, allodynia, and temporal summation to tonic pain [4,8,9,10]. In fact, preserved STT function, which has been characterized in the majority of patients with NP following clinically sensory complete SCI, could play an important role in the development of chronic SCI-NP, especially when continuously activated by pathophysiological mechanisms of segmental hyperexcitability and sensory input from dysreflexic pathways [11,12]. Indeed, neuroinflammation may trigger spontaneous activity in residual intact STT neurons that may constitute a spinal pain generator after a discomplete SCI [11], leading to severe NP [11,13,14]. Sensory discomplete SCI corresponds to a clinically complete lesion with neurophysiological evidence of residual translesional and infra-lesional sensorimotor connectivity [15,16,17]. Dysfunction of local and descending pain modulation mechanisms could explain the existence of NP in SCI (SCI-NP) due to hyperexcitability of residual function of the STT, contributing to severe NP [11,13,14]. In fact, early temporal summation to repeated pinprick stimulation, allodynia, and deficient endogenous pain modulation (EPM) have all been observed with the development of SCI-NP [3,4,9,18].

Increased neuronal excitability is considered a key feature of central sensitization to pain. Temporal summation of pain (TSP), which is described as increased pain perception in response to repeated or prolonged noxious stimulation [19,20], is considered as an important proxy of neuronal hyperexcitability, reflecting a pronociceptive mechanism [20]. Previous studies found contradictory findings for individuals with SCI related to TSP with tonic thermal stimuli [3,18,21,22]. Additionally, just one study [20] used the cold pressor test (CPT) applied at the hand or foot, but no differences were observed between individuals with or without SCI-NP [20].

Dysfunction of EPM has been identified in several chronic pain conditions [23,24,25,26,27] and animal models of SCI, which showed pathophysiological mechanisms of descending excitability and pronociception [28,29]. In humans, EPM is commonly assessed with the conditioned pain modulation (CPM), a psychophysical paradigm involving the application of a noxious test stimulus (TS), which is modulated by another remotely applied noxious conditioning stimulus (CS) [30,31,32,33] involving the activation of the spino-bulbo-spinal loop [23,32,33], although propriospinal control systems cannot be excluded [34]. Hence, dysfunction of EPM, i.e., impaired pain inhibition/diminished CPM efficiency, may contribute to the development and maintenance of NP after SCI [18,21,33,35]. Indeed, a spectrum of both antinociception and pronociception have been observed with SCI-NP [21]. Previous studies have observed deficient pain inhibition in SCI-NP compared with those without NP and healthy controls [18,21]. A longitudinal study showed a preservation of EPM with a progressive reduction in CPM efficiency in SCI-NP over time [35]. Moreover, CPM efficiency has also been related to NP characteristics (e.g., intensity and area): higher number of painful body regions and more intense NP were associated with deficient pain inhibition in SCI-NP [18,20,21]; however, more intense NP has also correlated with greater pain inhibition [33,35]. Hence, altered EPM may depend on the NP severity in individuals with SCI, but other factors such as SCI grade and chronicity have not been evaluated using translesional CPM test paradigms.

Although sensory hypersensitivity, including cold-evoked pain [36], might identify predictors of development of NP in the early stages of SCI [9,35,37], currently no studies demonstrating TSP and CPM-induced pronociception above and below sensory complete and incomplete SCI during the subacute and chronic phases of injury have been conducted. Thus, there is an urgent need to understand the mechanisms contributing to spinal hyperexcitability and EPM for an effective management and diagnostic guide for intervention [35]. The main aim of this study was to characterize TSP with the CPT and CPM-induced pronociception in individuals with SCI and NP compared with those without NP, with careful consideration of the stage (chronic/subacute) and severity (complete/incomplete) of SCI. Additionally, a secondary aim was to explore TSP and CPM according to the phenotype of NP (at-level/below-level), considering changes according to the level of injury.

## 2. Materials and Methods

This study protocol was approved by the local clinical research ethics committee (approval number 684; 2021) and conducted according to the Declaration of Helsinki [38]. Written informed consent was provided by all subjects before their inclusion in the study. Three experimental groups were included: healthy subjects (noninjured group), individuals with SCI and neuropathic pain (SCI-NP), and those without neuropathic pain (SCI-noNP). The exclusion criteria for the noninjured group (healthy individuals) included presence of trauma to the central or peripheral nervous system, previous or actual symptoms or signs of neurological disease or chronic pain, pain present during movement, inflammation, or local tissue damage.

### 2.1. Subjects with SCI

The classification of SCI, including neurological level of SCI, was evaluated following the International Standards for Neurological Classification of SCI and the American Spinal Cord Injury Association Impairment Scale (AIS) [39]. The clinical team recruited individuals aged between 18 and 85 years with SCI from a national reference hospital (Spain). The recruitment period extended from February 2021 to December 2022.

A neurological level of injury located between C5–T12, AIS A–D, and nontraumatic or traumatic etiology were the inclusion criteria for individuals with SCI. The presence of daily non-evoked pain and/or evoked pain during the previous 8 weeks were the inclusion criteria for NP. The diagnosis for central NP was performed following the classification of the NP subtype (i.e., below-level/at-level) according to the International Spinal Cord Injury Pain criteria, which were also applied for diagnosis of NP [40]. In fact, the diagnosis of NP was determined according to the grading system for definitive NP [16,41,42]. Furthermore, the pain intensity experienced during the preceding week, rated on the 11-point numerical rating scale (NRS, *see below*), was also considered. The individuals with NP were included in the study if they were following a constant 2-week medication for pain with antidepressants (amitriptyline), opioids (tramadol), antiepileptics (pregabalin or gabapentin) or anxiolytics (lorazepam), or even untreated. Nevertheless, brain injury, presence of altered sensation in the hand and face, cauda equina injury, fibromyalgia syndrome, history of chronic pain prior to SCI, autonomic dysreflexia, cognitive deficits, skin lesions overlying the V2, C6, and L4 dermatomes sensory testing sites according to the International Standards for Neurological Classification of Spinal Cord Injury (ISNCSCI) [43], or pain related to peripheral nervous system injury or neuropathy associated with pain evoked during movement, inflammation, or local tissue damage were the exclusion criteria for individuals with SCI [16,21]. Individuals who presented mild pain with a pain intensity rated less than 2 on the NRS (*see below*), in addition to less than 4 NP characteristics identified with the Douleur Neuropathique 4 questions (DN4, *see below*) were not considered for inclusion in the study.

Accordingly, in addition to 24 healthy individuals (41.4 ± 15.4 years, n = 24, 16 women), 48 individuals were finally recruited and assigned to the SCI-noNP group (53.2 ± 17.2 years, n = 24, 5 women, Table 1) and the SCI-NP group (48.8 ± 10.7 years, n = 24, 9 women, Table 2). Basic clinical and demographic characteristics (*see details below in Results section*) were collected as recommended in the International Spinal Cord Injury Core Data Set (Version 2.0) [44].

### 2.2. Experimental Sessions

**Figure 1:**
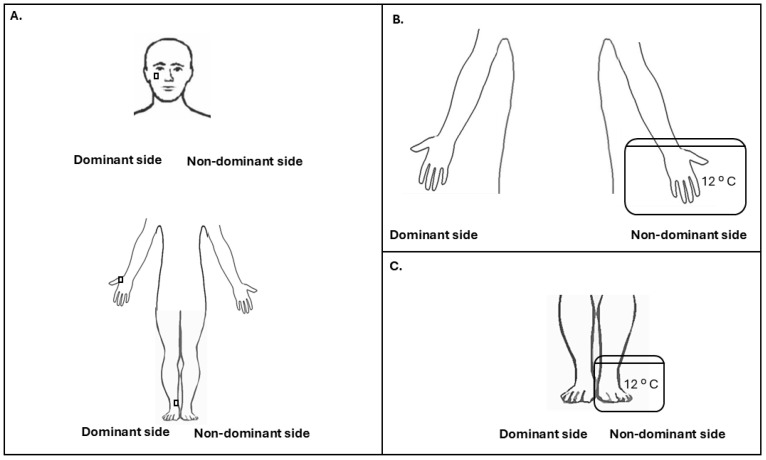
Test and conditioning stimuli. (**A**) Test stimuli (TS) application: pressure pain thresholds (PPTs) were assessed over several musculoskeletal structures on the dominant side: zygomatic bone (V2 dermatome), dorsal surface of the proximal phalanx of the thumb (C6 dermatome, AIS Key Sensory Point), and medial malleolus (L4 dermatome, AIS Key Sensory Point) before and after conditioning stimulus 1 (CS 1: hand immersion) and conditioning stimulus 2 (CS 2: foot immersion); (**B**) conditioning stimulus 1 (CS 1, cold pressor test (CPT) related to hand immersion): after TS, non-dominant hand was immersed wide open up to the wrist in a 15 L cold water bath (12 °C; 30 × 25 × 25 cm) for one minute; (**C**) conditioning stimulus 2 (CS 2, cold pressor test (CPT) related to foot immersion): after TS, non-dominant foot was immersed up to the ankle in a 15 L cold water bath (12 °C; 30 × 25 × 25 cm) for one minute.

All subjects participated in a 45-minute session performed in a quiet and temperature-controlled room. Participants sat in a relaxed manner on a comfortable chair and looked straight ahead. The session started with the pressure test stimulus (TS, *see below*) on the dominant side (Figure 1A). The cold pressor test (CPT) conditioning stimulus (CS, *see below*) was then applied to the non-dominant hand (Figure 1B), and the subject was asked to rate the tonic cold pain intensity during the heterotopic stimulus. After CS, the TS was applied on the dominant side (Figure 1A). A fifteen-minute break followed by application of the CS to the non-dominant foot was then performed (Figure 1C), and the subject was also asked to rate the tonic cold pain intensity during the heterotopic stimulus. After CS, the TS was also applied to the dominant side (Figure 1A). Thus, the study consisted of a sequential CPM paradigm, as recommended by a consensus group [32]; that is, all assessments were performed before (pre-conditioning) and after (post-conditioning) CPT of the hand or foot as the CS. Subjects were blinded to the study hypothesis and temperatures of the painful stimulus.

#### 2.2.1. Pain Assessment

The International Spinal Cord Injury Pain (ISCIP) Basic Data Set was used for the clinical pain assessment, as it is a questionnaire evaluating the pain area, type of pain, and its intensity, rated on an 11-point numerical rating scale (NRS; 0: no pain; 10: maximum pain), during the previous week [45]. Additionally, the DN4 screening questionnaire was used as a tool to confirm the previous diagnosis of NP [46]. The DN4 consists of 10 items (NP characteristics): 7 items (NP descriptors or neuropathic features) associated with the quality of pain (painful cold, burning, electric shocks) and their relationships with altered sensations (itching, numbness, pins and needles, tingling), and 3 items associated with neurological assessment in the painful area (pinprick hypoesthesia, touch hypoesthesia, tactile allodynia) [47]. Each positive (yes) item was scored as 1; therefore, the sum of the 10 items corresponds to the total DN4 score. For the diagnosis of NP, the cutoff value was a total score of DN of ≥4 of 10 [46,47].

In general, although some phenotypical differences have been reported between at-level and below-level pain [37], both types of pain were classified as NP.

#### 2.2.2. Test Stimulus (TS): Pressure Pain Threshold (PPT)

Pressure pain threshold (PPT) is defined as the minimal amount of pressure where the sensation of pressure changes to pain [48]. PPTs were assessed with an electronic algometer (FPIX^TM^, Wagner Instruments, Greenwich, CT, USA) over different musculoskeletal structures on the dominant side: zygomatic bone (V2 dermatome), dorsal surface of the proximal phalanx of the thumb (C6 dermatome, AIS Key Sensory Point) [39], and medial malleolus (L4 dermatome, AIS Key Sensory Point) [39] (Figure 1A). These sites were tested to determine generalized pain modulation following CS [32].

Testing was performed using standardized instructions in accordance with the protocol of the German Research Network on Neuropathic Pain [49]. Pressure was applied at a rate of approximately 5 Newtons (N) per second on each point [49,50]. The average of the 3 trials on each point, with 10 s interstimulus intervals, was recorded and used for the main analysis [50]. Excellent intra-session reliability for CPM assessment with PPTs has been reported [51,52].

#### 2.2.3. Conditioning Stimulus (CS): Cold Pressor Test

The cold pressor test (CPT) was chosen as the CS, since it has been shown to evoke CPM effect effectively and reliably in combination with varied test stimuli [51,53]. Subjects immersed their non-dominant hand wide open up to the wrist or non-dominant foot up to the ankle in a 15 L cold water bath (12 °C; 30 × 25 × 25 cm) [23] for one minute (Figure 1B,C), as 12 °C and the one-minute duration of the CS have been shown to be sufficient to evoke a CPM effect [52,54]. Temperature was controlled with continuous monitoring with a thermometer during the experiment. Pain ratings associated with cold water were obtained during immersion at 10 s intervals and immediately thereafter using an NRS (0: no pain; 10: maximum pain) [55] to determine tonic cold pain intensity during CPT. If the perceived pain became unbearable, subjects were allowed to remove their foot or hand at any time [56]. The calculation of net CPM using a control for the CPT presented at 32 °C [21] was not included in this protocol, given that poor–fair reliability has been identified with the use of the control condition [57]. Temporal Summation of Pain (TSP) was calculated by subtracting the last NRS rating from the first [3,58]. Negative values show pain habituation, and positive values symbolize pain summation [20]. Percentage normalization of temporal summation was not adopted in this study, as the evoked pain intensity at 10 s during the foot CPT was 0 in the complete SCI group without NP.

The CPM was quantified by comparing PPTs (TS) performed before and after the CPT. The percentage difference between TS values after and before CS (CPM = (TS2 − TS1) × 100/TS1) determined the magnitude of CPM effects [35]. Positive values underscore the existence of CPM-induced antinociception (increase in PPT, pain inhibition), while negative values show CPM-induced pronociception (decrease in PPT, pain facilitation) [32,59].

### 2.3. Statistical Analysis

Data analysis was conducted using SPSS version 22.0 (IBM Corp., Armonk, New York, NY, USA) and Sigma Plot version 12.0 (Systat Software, Inc., San Jose, CA, USA). Results are expressed as mean and standard deviation (SD), percentages, or median with interquartile range (IQR). The Kolmogorov–Smirnov test was considered for the normal data distribution. Demographic and clinical characteristics of both SCI groups (SCI-NP, SCI-noNP) were compared using the Mann–Whitney U test and χ^2^ tests of independence. For intergroup comparisons (*groups:* noninjured, SCI-NP, SCI-noNP; *subgroups according to stage of SCI*: subacute SCI-NP, subacute SCI-noNP, chronic SCI-NP, chronic SCI-noNP; *subgroups according to severity of SCI:* complete SCI-NP, incomplete SCI-NP, complete SCI-noNP, incomplete SCI-noNP; *subgroups according to stage and severity of SCI*: chronic complete SCI-NP, chronic incomplete SCI-NP, chronic complete SCI-noNP, chronic incomplete SCI-noNP, subacute complete SCI-NP, subacute incomplete SCI-NP, subacute complete SCI-noNP, subacute incomplete SCI-noNP), Kruskal–Wallis one-way analysis of variance test was performed, followed by the Dunn’s post hoc test. The Mann–Whitney U test was only used for comparisons between two selected subgroups from the SCI group. The Friedman test followed by post hoc pairwise comparisons with the Wilcoxon matched-pairs signed rank test were performed in order to investigate TSP during the CPT (trend of pain across time). The changes in DN4 descriptors related to hand or foot immersion were assessed with the Wilcoxon signed-rank test. The CPM effect on PPT was also assessed with the Wilcoxon signed-rank test. Furthermore, Spearman’s rho (rs) was used to evaluate associations between variables. Finally, due to phenotypical differences between at-level and below-level NP [37], an additional exploratory subanalysis considering at-level and below-level pain, following the statistical tests previously described, was also performed in the SCI-NP group and subgroups (stage, severity) for a comprehensive NP analysis. Additionally, due to possible influence of sex in pain modulation [60], a general additional secondary analysis considering the sex variable in CPM effect for both SCI-NP and SCI-noNP groups was also performed. A 95% confidence level was considered for the statistical analysis. A *p*-value < 0.05 was considered statistically significant.

## 3. Results

### 3.1. Demographic and Clinical Characteristics

A total of 72 participants were recruited: 48 individuals with SCI (SCI-NP: n = 24; SCI-noNP: n = 24) and 24 healthy controls (noninjured group). Demographic and clinical characteristics of the subjects with SCI are presented for both no pain (SCI-noNP, Table 1) and NP groups (SCI-NP, Table 2). No significant difference was identified for the mean age of participants between noninjured (41.4 ± 15.4 years, n = 24, 16 women), SCI-noNP (53.2 ± 17.2 years, n = 24, 5 women) and SCI-NP (48.8 ± 10.7 years, n = 24, 9 women; *p* > 0.05). Furthermore, no significant difference was found in the time since SCI between SCI-noNP and SCI-NP (Table 1 and Table 2; *p* > 0.05). Both SCI groups included subjects with complete (AIS A: n = 12) and incomplete (AIS B-D: n = 12) SCIs and neurological levels ranging from C5 to T12 in subacute (n = 12) or chronic (n = 12) periods (Table 1 and Table 2).

In the SCI-NP group, evoked NP was identified only at-level (n = 4), only below-level (n = 17), and both at-level and below-level (n = 3, Table 2). The mean intensity for NP was 6.5 ± 2.2, while the mean intensity at-level NP, below-level NP, and both at-level and below-level NP were 6.7 ± 2.5, 6.2 ± 2.2, and 7.5 ± 2.5, respectively (Table 2). Examination of the NP characteristics in the SCI-NP group with DN4 revealed a mean score of 5.6 ± 1.6 (Table 2).

Significant associations between pain intensity and clinical variables were found in the SCI group. The 7-day pain intensity was significantly and positively associated with pain duration (rho = 0.687; *p* < 0.001; Figure S1A) and DN4 total score (rho = 0.833; *p* < 0.001; Figure S1B): a greater number of NP characteristics and longer pain duration were associated with higher pain intensity.

Results from baseline mechanical TS revealed no significant differences between SCI groups (*p* > 0.05). Nevertheless, in general, significant sex differences in PPTs over V2 and C6 dermatomes were found in SCI (*p* < 0.03; Figure S1C): women exhibited lower PPTs than men in these points (V2: 16.7 ± 6.4 vs. 22.4 ± 6.1; C6: 29.3 ± 11.3 vs. 35.2 ± 9.8).

### 3.2. CPM Effect

#### 3.2.1. CPT: Hand Immersion

**Figure 2:**
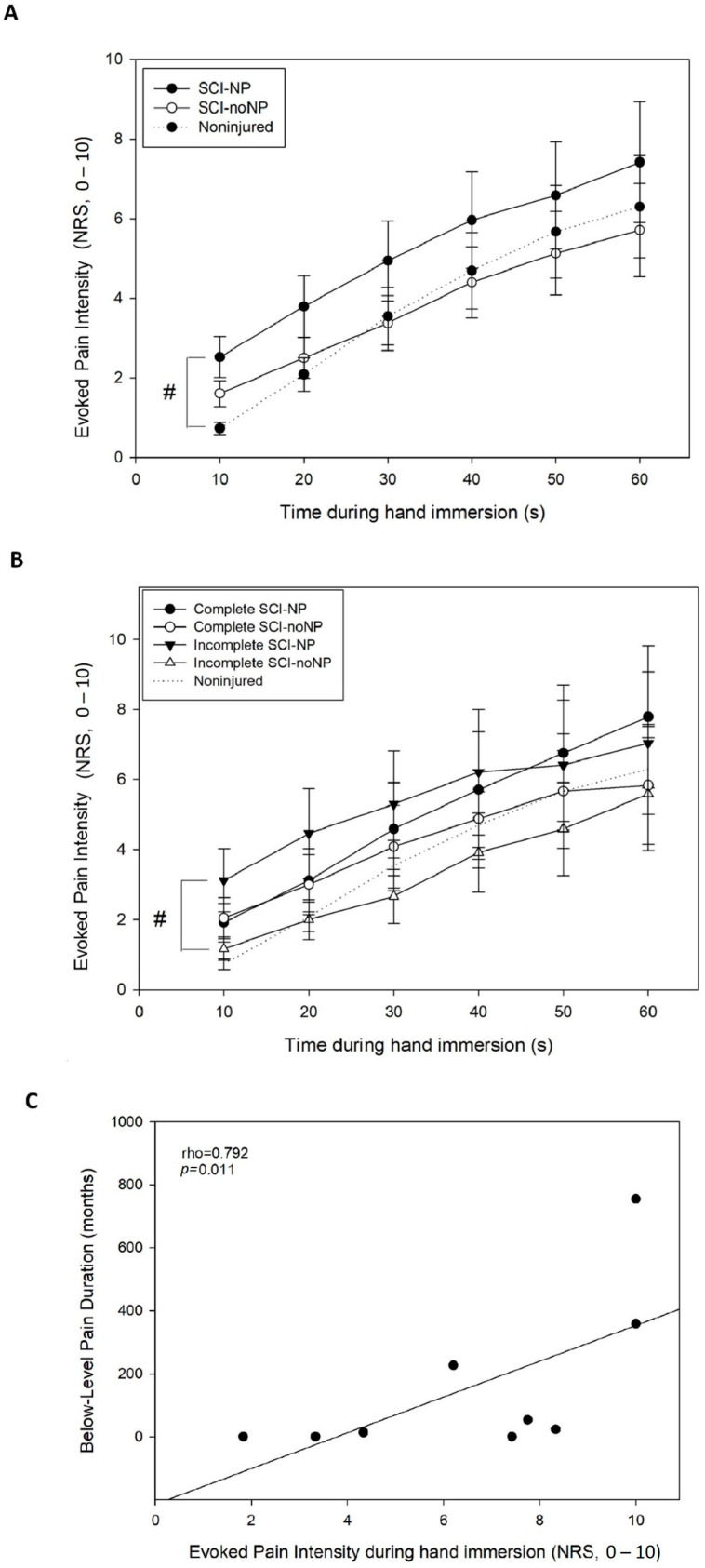
Mean evoked cold pain intensity during hand immersion with the cold pressure test (60 s) in individuals with spinal cord (SCI) with or without NP and healthy participants. (**A**) Mean evoked pain intensity during cold water immersion (12 °C) in individuals with SCI with or without neuropathic pain (SCI-NP or SCI-noNP, respectively) and noninjured (healthy controls); (**B**) mean evoked pain intensity during cold water immersion (12 °C) in individuals with complete or incomplete SCI and presence or absence of neuropathic pain (SCI-NP or SCI-noNP, respectively); (**C**) association between evoked cold pain intensity during hand immersion and duration of below-level NP. *NRS: numerical rating scale. # p < 0.05 (intergroup comparisons). Data are shown as mean and SE (standard error)*.

Maximal pain ratings during cold water immersion were found in the SCI-NP group at 60 s (Figure 2A), specifically in chronic complete SCI-NP (8.1 ± 3.7). The gradual increase in pain intensity during hand immersion was detected in all groups (Figure 2A). The Friedman test revealed a significant global effect of time on the pain ratings during tonic cold stimulation (TSP) in all groups (*p* < 0.001). In the SCI-NP, SCI-noNP, and noninjured groups, post hoc pairwise comparisons showed a significant effect of time on pain ratings during tonic cold stimulation (TCS) at 20, 30, 40, 50, and 60 s (SCI-NP: *p* < 0.001; SCI-noNP: *p* < 0.001; noninjured: *p* < 0.001).

The largest changes in pain intensity during hand immersion were found in SCI-NP (Figure 2), especially in subacute complete and chronic complete SCI-NP. Furthermore, significant differences between the SCI-NP and noninjured groups were found: SCI-NP showed greater increase in pain intensity (TSP) than that in noninjured (*p* = 0.03; Figure 2A). Additionally, significant differences between incomplete SCI-NP and incomplete SCI-noNP were found: incomplete SCI-NP showed greater increase in pain intensity (TSP) than that in incomplete SCI-noNP (*p* = 0.04; Figure 2B). No other significant differences between groups or subgroups were found.

Finally, the pain intensity during hand immersion was significantly and positively associated with below-level NP duration (rho = 0.792, *p* = 0.011; Figure 2C). No other significant associations between pain intensity or TSP during hand immersion and clinical variables were found.

#### 3.2.2. CPT: Foot Immersion

**Figure 3:**
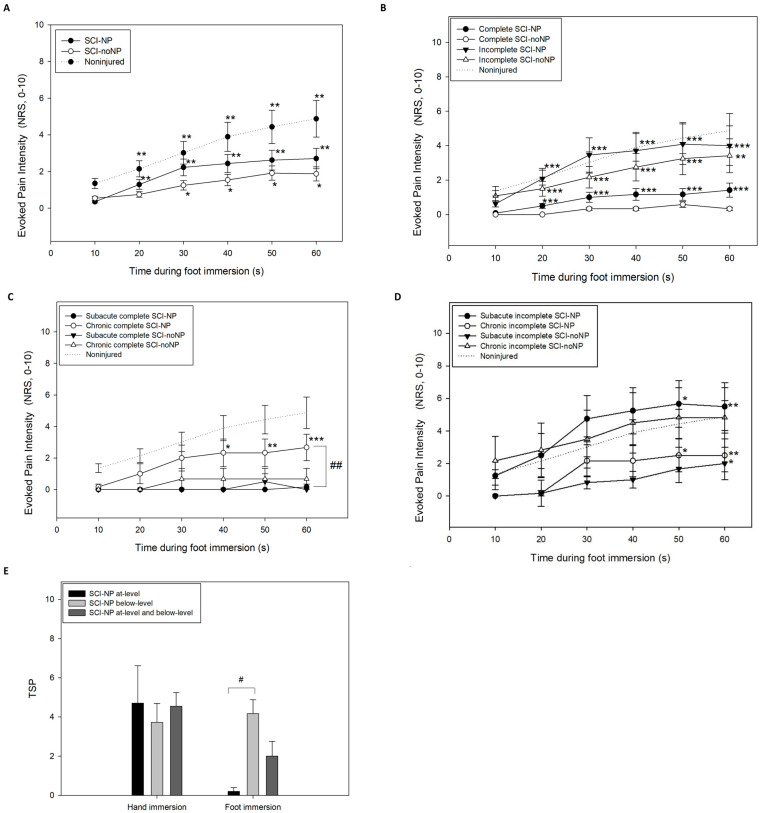
Mean evoked cold pain intensity during foot immersion with the cold pressure test (60 s) in individuals with spinal cord injury (SCI) and healthy participants. (**A**) Mean evoked cold pain intensity during cold water immersion (12 °C) in individuals with SCI with or without neuropathic pain (SCI-NP or SCI-noNP, respectively) and noninjured (healthy controls); (**B**) mean evoked cold pain intensity during cold water immersion (12 °C) in individuals with complete or incomplete SCI and presence or absence of neuropathic pain (SCI-NP or SCI-noNP, respectively); (**C**) mean evoked cold pain intensity during cold water immersion (12 °C) in individuals with complete SCI and presence or absence of neuropathic pain (SCI-NP or SCI-noNP, respectively) in subacute or chronic period of SCI; (**D**) mean evoked cold pain intensity during cold water immersion (12 °C) in individuals with incomplete SCI and presence or absence of neuropathic pain (SCI-NP or SCI-noNP, respectively) in subacute or chronic period of SCI; (**E**) temporal summation of cold pain (TSP) intensity associated with foot and hand immersion with the cold pressor test (12 °C) in SCI with at-level, below-level, and both at-level and below-level neuropathic pain (NP). NRS: numerical rating scale. * p < 0.05; ** p < 0.01; *** p < 0.001 (intragroup comparisons). # p < 0.05; ## p < 0.01 (intergroup comparisons). Data are shown as mean and SE (standard error).

The maximal pain ratings during cold water immersion were found in SCI-NP (Figure 3), specifically in subacute incomplete SCI-NP at 50 s (5.7 ± 3.5; Figure 3D), followed by chronic incomplete SCI-noNP (4.8 ± 4.5; Figure 3D), noninjured (4.9 ± 3.1; Figure 3), and chronic complete and chronic incomplete SCI-NP (2.7 ± 2.1 vs. 2.5 ± 2.5; Figure 3C,D) at 60 s.

A gradual increase in pain intensity during foot immersion was detected in all groups (Figure 3). The Friedman test revealed a significant global effect of time on the pain ratings during TCS (TSP) in all groups (*p* < 0.001). In the SCI-NP and the noninjured groups, post hoc pairwise comparisons showed a significant effect of time on pain ratings during TCS at 20, 30, 40, 50, and 60 s (SCI-NP: *p* < 0.001; noninjured: *p* < 0.001; Figure 3A). In SCI-noNP, post hoc pairwise comparisons revealed a significant effect of time on pain ratings during TCS at 30, 40, 50, and 60 s (*p* < 0.02; Figure 3A). Nevertheless, post hoc pairwise comparisons showed no significant effect of time on pain ratings during TCS in complete SCI-noNP (*p* > 0.05; Figure 3B).

Significant differences between SCI and noninjured were found in TSP and maximal pain intensity during CPT (*p* < 0.05). Significant differences between subacute complete SCI-NP and chronic complete SCI-NP at 30, 40, 50, and 60 s were found (*p* < 0.02; Figure 3C): chronic complete SCI-NP exhibited higher pain intensity than that in subacute complete SCI-NP. In contrast, subacute incomplete SCI-NP showed higher pain intensity than that reported in either subacute incomplete SCI-noNP (*p* = 0.04) or chronic incomplete SCI-NP (*p* < 0.01; Figure 3D). Finally, significant differences between below-level NP and at-level NP were found (*p* < 0.03; Figure 3E): individuals with below-level NP showed higher pain intensity and TSP during foot CPT compared with those with only at-level NP. No other significant differences between groups or subgroups were found.

No significant associations between pain intensity or TSP during foot immersion and clinical variables were found (*p* > 0.05).

#### 3.2.3. Translesional CPT-Induced Pronociception Above and Below the SCI

**Figure 4:**
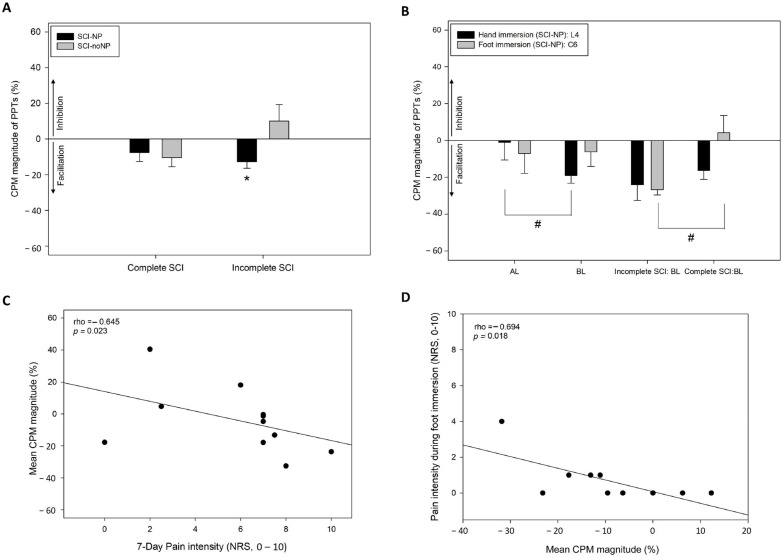
Conditioned pain modulation (CPM) of pressure pain thresholds (PPTs) following application of the cold pressor test (CPT) to the hand and foot in individuals with spinal cord injury (SCI) and presence or absence of neuropathic pain (SCI-NP or SCI-noNP, respectively). (**A**) CPM magnitude of PPTs measured at the L4 dermatome following hand CPT; (**B**) CPM magnitude of PPTs measured at the L4 and C6 dermatomes following hand and foot CPT (respectively) in individuals with SCI-NP at-level or below-level the injury; (**C**) association between mean CPM-induced change in PPT measured at the V2, C6, and L4 dermatomes following hand CPT and non-evoked (spontaneous) 7-day pain intensity in individuals with incomplete SCI-NP; (**D**) association between mean CPM-induced change in PPT at the V2, C6, and L4 dermatomes following foot CPT and evoked cold pain intensity during foot immersion in individuals with complete SCI-NP. AT: At-level neuropathic pain; BL: below-level neuropathic pain; NRS: numerical rating scale. * p < 0.05 (significant intragroup CPM effect). # p < 0.05 (intergroup comparisons). Data are shown as mean and SE (standard error).

After hand immersion, CPT-induced pronociception (pain facilitation) measured at the L4 dermatome as a decrease in PPT was detected in incomplete SCI-NP (−12.7%; *p* = 0.015; Figure 4A). Furthermore, significant differences between at-level NP and below-level NP (−1% vs. −19%, *p* = 0.019; Figure 4B) or below-level NP and both at-level and below-level NP (−19% vs. −5%, *p* = 0.047; Figure 4B) on the PPT over L4 dermatome were identified. No other significant differences between groups or subgroups were identified.

After foot immersion, the CPT induced significant pronociception measured at the C6 dermatome in the incomplete SCI-NP group with below-level NP compared to the group with complete SCI below-level NP (−27% vs. 4%; *p* < 0.05; Figure 4B). No other significant differences between groups or subgroups were identified.

Significant correlations were found between 7-day (spontaneous) non-evoked pain intensity and whole-body pronociception, measured at the V2, C6 and L4 dermatomes, induced with the hand CPT in the incomplete SCI-NP subgroup (rho = −0.645; *p* = 0.023; Figure 4C): a higher pain intensity was associated with pronociception after the hand CPT. Additionally, a significant association between evoked pain intensity during the foot CPT and whole-body pronociception was found in the complete SCI-NP group (rho = −0.694; *p* = 0.018; Figure 4D): a higher evoked pain intensity during the foot CPT was associated with greater pronociception after foot immersion.

In general, no significant sex differences in CPM effect were found in SCI. Nevertheless, significant sex differences were found in PPTs over the C6 dermatome after hand immersion (*p* = 0.035) and PPTs over the V2 dermatome after the hand (*p* = 0.002) and foot (*p* = 0.01) CPTs in SCI: women exhibited lower PPTs than men in these points (hand: V2: 15.7 ± 4.9 vs. 22.6 ± 8.1; C6: 27.3 ± 7.4 vs. 33.3 ± 10.1; foot: V2: 16.2 ± 5.6 vs. 26.5 ± 11.4).

#### 3.2.4. CPT-Evoked Neuropathic Features

Neuropathic features described in the DN4 questionnaire were also evoked by hand and foot immersion (Supplementary Tables and Figures).

Neuropathic features during hand immersion were observed in 62.5% of individuals with SCI (SCI-noNP: 54.1%; SCI-NP: 70.8%) and 58.3% of healthy subjects (noninjured). In fact, a significant increase in the number of descriptors during hand immersion was revealed in all groups (*p* < 0.001). Significant differences between noninjured and SCI-NP were found (*p* = 0.008).

During hand immersion, the most common descriptor was painful cold in all groups (SCI-NP: 77%; SCI-noNP: 76.5%; noninjured: 57.1%; Figure S2A; Tables S1 and S2). Nevertheless, the number of neuropathic features was significantly higher in chronic SCI than in subacute SCI within the SCI groups (*p* < 0.05), especially in the incomplete SCI-NP subgroup (*p* = 0.009). Furthermore, an additional subanalysis revealed that individuals with SCI-NP and the highest scores in pain intensity (NRS > 5) showed higher number of neuropathic features than those with SCI-NP and lower scores in pain intensity (NRS ≤ 5) (*p* < 0.05). No other significant differences between groups or subgroups were found.

During PPT assessment after hand immersion, the SCI-NP (54.2%) and SCI-noNP (41.7%) groups showed neuropathic features, highlighting electric shocks as the most common neuropathic features (Figure S2B; Tables S1 and S2). The number of neuropathic features was significantly higher in incomplete SCI than in complete SCI within the SCI groups (*p* < 0.05). No other significant differences between groups or subgroups were found.

Neuropathic features during foot immersion were observed in 50% of individuals with SCI (SCI-NP: 70.8%; SCI-noNP: 29.2%; Figure S2C; Tables S3 and S4) and 4.2% of healthy subjects (noninjured). In fact, a significant increase in the number of descriptors during foot immersion was revealed in all groups (*p* < 0.001). Significant differences between groups were found (*p* < 0.02), particularly between SCI-noNP and SCI-NP (*p* < 0.01) and between noninjured and SCI-NP (*p* = 0.006). No other significant differences between subgroups were found.

During foot immersion, the most common descriptor found was painful cold (SCI-NP group: 31.2%; SCI-noNP group: 71.4%; noninjured group: 100%) and tingling (SCI-NP group: 43.7%; Figure S2C; Tables S3 and S4). Nevertheless, the number of neuropathic features was significantly higher in SCI-NP than in SCI-noNP (*p* < 0.05). No other significant differences between groups or subgroups were found.

During PPT assessment after foot immersion, the SCI-NP (70.8%) and SCI-noNP (25%) groups showed neuropathic features, highlighting electric shocks (SCI-noNP: 50%; SCI-NP: 30%) and tingling (SCI-NP: 40%) as the most common neuropathic features in SCI-noNP and SCI-NP, respectively (Figure S1D; Tables S3 and S4). The number of neuropathic features was significantly higher in the SCI-NP group than in the SCI-noNP group (*p* < 0.05). Additionally, the number of neuropathic features was significantly higher in the complete SCI-NP subgroup than in the noninjured group (*p* < 0.05), especially in the chronic complete SCI-NP subgroup compared with the noninjured group (*p* < 0.02). No other significant differences between groups or subgroups were found.

## 4. Discussion

This is the first study showing differences in CPM and tonic cold perception in individuals with SCI with and without NP according to the stage and severity of SCI. In fact, this study reveals for the first time differences in tonic cold perception and translesional CPM-induced pronociception in individuals with SCI with NP above and below sensory complete and incomplete injury, in addition to the association of these possible pathophysiological mechanisms with spontaneous pain intensity and below-level NP. Although TSP during TCS was observed in all groups, chronic complete SCI-NP and subacute incomplete SCI-NP showed the highest pain intensity and the biggest changes in pain intensity (TSP) during the hand and foot CPTs, respectively. Indeed, individuals with below-level NP showed higher TSP during foot immersion compared with those with at-level NP. Furthermore, incomplete and complete SCI-NP demonstrated pronociception after hand and foot immersion, respectively. Non-evoked 7-day pain intensity was associated with whole-body pronociception after CPT in the incomplete SCI-NP group, highlighting individuals with SCI and below-level NP.

### 4.1. TSP and Pronociception Induced by TCS

Although TSP during TCS was observed in all SCI subgroups, chronic complete SCI-NP and subacute incomplete SCI-NP showed the highest evoked cold pain intensity and the biggest changes in pain intensity during the hand and foot CPTs, respectively. Previous studies also found increased TSP during tonic heat noxious stimulation in SCI-NP [18,21]; however, no differences between SCI-NP and healthy controls were previously shown [3,20,22], unlike this research. Nevertheless, TSP protocols using repetitive phasic noxious stimulations at/below-level SCI have demonstrated enhanced TSP in SCI-NP compared with control groups [61,62,63]. In this study, individuals with below-level NP showed higher TSP during the foot CPT compared with subjects with at-level NP, which may support the association between cold hypersensitivity and below-level NP, in addition to phenotypical differences between at-level and below-level NP, as previously reported [37]. The current study also reveals that individuals with chronic complete SCI-NP reported a bigger change in evoked pain intensity compared with the subacute complete SCI-NP group during the foot CPT. In fact, previous findings might support these results as below-level NP develops in the first year postinjury and is often associated with more severe symptoms [7,64]. Furthermore, a much larger proportion of individuals with a more severe injury reported NP compared with those with less severe symptoms [64,65]. Additionally, residual STT function and hyperexcitability below injury have been found in discomplete SCI [11].

Although TSP above the level of injury has been previously revealed during tonic heat stimuli in SCI-NP [18,21], this is the first study demonstrating TSP during TCS both above and below level, which supports the presence of a generalized neuronal hyperexcitability in SCI-NP [18]. Increased TSP above level has been associated with spontaneous below-level NP [18], as altered processing of somatosensory function in dermatomes rostral to segmental injury level has been previously identified in SCI-NP [66].

Previous studies have reported neuronal hyperexcitability at/below-level SCI-NP, detected by allodynia, hyperpathia, or mechanical windup pain, and associated with alterations in STT function [8,36,62,67]. In fact, dysfunction of STT may be resulting from development of local inflammatory factors within the spinal cord, dysreflexia pathways or development of descending facilitatory pathways [28,29], or a decrease in descending inhibition, which could lead to hyperexcitability in residual nociceptive neurons and, hence, generalized central sensitization, hyperexcitability, and spontaneous pain (NP) [18]. Higher TSP in the chronic sensory complete SCI-NP group compared to the subacute group, and lower TSP in chronic incomplete SCI-NP compared to the subacute group suggest that local neuronal excitability, especially below the level of SCI, are dynamic and may depend on both injury severity and stage.

Pain facilitation (pronociception) for pressure stimuli induced by the CPT, applied as CS, and measured across injury site above and below level in sensory complete and incomplete SCI-NP were revealed for the first time. Previous studies have also shown pronociception in SCI-NP [18,21], including development of at-level EPM dysfunction during early stages of SCI for SCI-NP [3]. Furthermore, this is the first study showing that individuals with below-level NP demonstrated greater pronociception compared with those with at-level NP. These findings advance our most recent understanding of clinical concepts of SCI-NP, including an association between below-level NP and reduced descending pain inhibition [18], neural hyperexcitability (hypersensitivity) prior to central pain and below-level central pain development [9], and differences in EPM dysfunction between at-level and below-level NP. In fact, although a possible reduction in descending inhibition at the SCI level was previously observed in SCI-NP [3,4], below-level NP is considered a central pain caused by spinal cord damage while at-level NP can be caused by lesions in nerve roots and/or the spinal cord [37], which could explain these results. In contrast, a greater pronociception with higher 7-day pain intensity in incomplete SCI-NP was shown, similar to previous results [21]. Additionally, this is the first study reporting greater CPT-induced pain facilitation associated with higher evoked cold pain intensity during foot immersion in clinically sensory complete SCI with NP, which could suggest the presence of residual STT function (discomplete SCI) that may also contribute to severe NP [11,14]. These findings may also suggest that the higher the spontaneous pain intensity, the more facilitatory or deficient the inhibitory EPM capacity, as previously reported [20]. Furthermore, pronociception above/below level also might suggest widespread central sensitization throughout neuroaxis at the spinal and/or supra-spinal levels [18] and compromised inhibitory EPM from early stages of SCI-NP [35]. A greater emphasis on understanding pronociceptive changes induced across the injury site may lead to a better understanding of segmental changes and hyperexcitability associated with development of SCI-NP.

### 4.2. Neuropathic Features Evoked by TCS

A greater number of neuropathic features evoked by the hand CPT were found in chronic SCI compared with subacute SCI, especially in the incomplete SCI-NP subgroup. Painful cold and tingling were the most common descriptors evoked by cold water immersion. In fact, individuals with SCI-NP and the highest pain intensity scores showed a greater number of neuropathic features evoked by hand immersion. Although an association between pain modulatory capacity and spontaneous NP characteristics was previously reported in SCI (i.e., less inhibitory EPM correlated with higher spontaneous burning pain intensity) [21], this is the first study reporting neuropathic features evoked by TCS and a relationship with duration of below-level SCI-NP, which supports hypothesis that spontaneous NP perception develops progressively after SCI [2,68]. These findings could be a result of either hyperactive ascending tracts [18] as a result of spinal hyperexcitability [36] and/or “pain generators” in thalamus [8,18] and cortex [18].

### 4.3. Influence of Sex on CPM Effect

Although women with SCI showed lower PPTs than men, no significant differences in CPM effect were found, as previously reported for chronic pain [69,70]. Nevertheless, greater pronociception after the hand CPT was found in women compared with men (L4 dermatome). These findings might be explained by sex differences in central nervous system pain processing (e.g., lower μ-opioid receptor binding activity in women) [52,71] and possible influence of menstrual cycle phases [23,52,72,73]. In fact, variations in pain responses due to fluctuating hormone levels could explain these differences, showing that women and men have comparable CPM during the ovulatory phase of women [74]. Thus, the complexity of CPM variability, which also includes personal attributes and attributes of the CS [60], may explain these discrepancies and prior unclear results [75].

Some limitations of this study should be considered. Firstly, one type of TS [32] was included, although the PPT and CPT are considered as the most reliable TS and CS for CPM (respectively) [32,52]. In fact, the selected temperature for CS (12 °C) has been shown to be well tolerated, especially in SCI-NP [21], avoiding violating the testing protocol [52]. Secondly, a sham condition may have been helpful to calculate the net CPM effect [32], although its use leads to poor–moderate reliability [57]. Furthermore, the CPT is considered as a well-validated test applied to different dermatomes [52,76], which reliably evokes CPM with different TS [51,52,53]. Additionally, a sequential CPM design has been used to avoid distraction bias [32], and a standardized manner to perform all measurements also reduced bias [69]. Thirdly, potential interference of analgesic medication was not controlled for [18,20].

These findings support an association between increased cold pain sensitivity and development of chronic pain in sensory discomplete/incomplete SCI [77], and suggest pain hyperexcitability and loss of top-down inhibitory control at [3,4]/below injury [11] from early stages [9,37]. In fact, ascending axons crossing injury may activate the propriospinal system [34] close to the lesion, facilitating the infra-lesional network [78] reflecting earlier evidence for functional correlation between at/below-level SCI-NP [36]. Furthermore, presence of evoked infra-lesional cold pain intensity, including in clinically complete chronic SCI-NP, might confirm preserved STT integrity [13], which could be influenced by dysfunction of descending inhibitory control resulting in hyperexcitability [11,13,14,18]. In contrast, a speculative interpretation of reduced infra-lesional TSP in chronic incomplete SCI-NP compared to the acute phase could reflect a re-establishment of descending inhibitory control. Although these findings support the potential role of differential activation of descending pronociceptive/antinociceptive processes, they cannot be used to conclude whether change in EPM is a cause/consequence of chronic NP and the impact of SCI stage and severity [20]. Nevertheless, this study provides a new view for understanding of how translesional pronociceptive processes may interact with residual infra-lesional STT hyperexcitability in clinically sensory complete SCI and development of below-level NP [11,12], which assists in correctly choosing analgesic treatment (personalized medicine approach) [79] and supports implementation of pretreatment measures preventing pain chronification [18] from early stages.

## 5. Conclusions

This study reveals significant differences in TSP between SCI and noninjured groups during the foot CPT, highlighting greater changes in pain intensity (TSP) in chronic complete SCI-NP and subacute incomplete SCI-NP. Furthermore, translesional CPM-induced pronociception was induced after hand CPT in the incomplete SCI-NP group. Individuals with below-level SCI-NP showed higher TSP during the foot CPT and greater hand CPT-induced translesional pronociception compared to at-level SCI-NP. Further understanding of changes in translesional inhibitory mechanisms and spinothalamic hyperexcitability according to SCI stage and severity assessed above/below injury should facilitate early personalized mechanism-based treatment. Further studies with larger sample sizes and longitudinal designs are now needed.

## Figures and Tables

**Table 1:** Demographic and clinical characteristics of participants with SCI without neuropathic pain (n = 24).

Subject Number	Gender	Age (Years)	AIS (A–D)	SCI Neurological Level	Etiology *	Time Since Injury (Months) #
#1	M	38	A	T4	T	1
#2	M	18	A	C5	T	1.5
#3	M	33	A	T8	T	2.5
#4	F	59	A	T4	NT	2.5
#5	M	59	A	T3	T	3
#6	M	67	A	T6	NT	4
#7	M	35	A	T4	T	6
#8	M	76	A	T11	NT	12
#9	M	51	A	T10	T	252
#10	M	66	A	T7	T	348
#11	M	51	A	T5	T	384
#12	M	61	A	T4	T	504
n = 12	11 M 1 F	51.2 ± 16.9	12 A	1 C 11 T	9 T 3 NT	126.7 ± 189.2
#13	F	26	B	C8	T	3
#14	M	69	B	C5	T	276
#15	F	41	C	T11	NT	1
#16	M	48	C	T12	NT	3.5
#17	F	84	C	T11	T	20
#18	M	26	C	T5	T	21
#19	M	53	C	T10	T	276
#20	M	71	C	T9	T	480
#21	M	69	D	T6	T	2
#22	M	66	D	T1	NT	2
#23	M	52	D	T9	NT	2
#24	F	58	D	T5	NT	420
n = 12	8 M 4 F	55.3 ± 17.9	2 B 6 C 4 D	2 C 10 T	7 T 5 NT	128.2 ± 181.9
n = 24	19 M 5 F	53.2 ± 17.2	24 A–D	3 C 21 T	15 T 8 NT	126.1 ± 182.3

Data are shown as mean ± SD or frequencies. AIS: American Spinal Injury Association Impairment Scale; F: female; M: male; NT: non-traumatic; T: traumatic; SCI: Spinal cord injury. * The etiology of traumatic SCI includes “motor vehicle/pedestrian accident” (n = 12), “other accident” (n = 3), “fall” (n = 1). The etiology of non-traumatic SCI includes “inflammation/infection” (n = 1), “transverse myelitis” (n = 2), “tumor” (n = 2), “vertebral column degenerative disorders” (n = 1), “vascular disorders” (n = 2). # Subacute SCI was considered in injuries less than 6 months (n = 12; complete SCI: n = 6; incomplete SCI: n = 6).

**Table 2:** Demographic and clinical characteristics of participants with SCI and neuropathic pain (n = 24).

Subject Number	Gender	Age (Years)	AIS (A–D)	SCI Neurological Level	Etiology *	Time Since Injury (Months) #	Pain Intensity (NRS: 0–10)	Area of Pain	DN4
#25	F	48	A	T5	T	1	7	Below level	4
#26	M	46	A	T11	T	1.5	7	At level	4
#27	M	38	A	T5	NT	2	6	At level	8
#28	M	26	A	T4	T	4	10	At/below level	9
#29	M	58	A	T1	T	5	5	At/below level	4
#30	M	47	A	T4	T	5	6	Below level	5
#31	M	61	A	T5	T	21	10	At level	5
#32	M	42	A	C7	T	28.5	4	At level	4
#33	F	22	A	T10	T	100	7.5	At/below level	7
#34	M	52	A	T8	T	228	6	Below level	7
#35	M	46	A	T5	T	360	6	Below level	4
#36	M	51	A	T4	T	372	8	Below level	8
n = 12	10 M 2 F	44.8 ± 11.6	12 A	1 C 11 T	11 T 1 NT	94 ± 143.1	6.9 ± 1.8	7 AT 8 BELOW	5.8 ± 1.9
#37	M	58	B	C5	T	240	10	Below level	6
#38	F	70	C	T10	T	2	2	Below level	4
#39	F	53	C	T11	NT	2	7	Below level	5
#40	M	49	C	T1	T	5.5	2	Below level	6
#41	F	54	C	T6	T	7	7	Below level	5
#42	F	51	C	T4	T	108	8	Below level	5
#43	M	48	D	C5	NT	1	7.5	Below level	6
#44	F	54	D	T10	NT	1.5	2.5	Below level	4
#45	F	40	D	T8	NT	2.5	7	Below level	5
#46	M	53	D	T4	T	96	7	Below level	9
#47	F	41	D	T4	NT	228	6	Below level	5
#48	M	63	D	T7	NT	756	7	Below level	6
n = 12	5 M 7 F	52.8 ± 8.4	1 B 5 C 6 D	2 C 10 T	6 T 6 NT	120.8 ± 218.9	6.1 ± 2.5	12 BELOW	5.5 ± 1.3
n = 24	15 M 9 F	48.8 ± 10.7	24 A–D	3 C 21 T	18 T 7 NT	107.4 ± 181.4	6.5 ± 2.2	7 AT 20 BELOW	5.6 ± 1.6

Data are shown as mean ± SD or frequencies. AIS: American Spinal Injury Association Impairment Scale; DN4: Douleur Neuropathique 4 screening questionnaire; F: female; M: male; NT: non-traumatic; T: traumatic; SCI: Spinal cord injury. * The etiology of traumatic SCI includes “motor vehicle/pedestrian accident” (n = 10), “surgical complication” (n = 2), “other accident” (n = 3), “fall” (n = 1). The etiology of non-traumatic SCI includes “tumor” (n = 4), “vertebral column degenerative disorders” (n = 1), “vascular disorders” (n = 1), “spinal dysraphism” (n = 1). # Subacute SCI was considered in injuries less than 6 months (n = 12; complete SCI: n = 6; incomplete SCI: n = 6).

## Data Availability

The original contributions presented in the study are included in the article/Supplementary Materials; further inquiries can be directed to the corresponding author/s.

## Supplementary Materials

The following supporting information can be downloaded at: https://www.mdpi.com/article/10.3390/healthcare12222300/s1, Figure S1: Clinical associations and pressure pain thresholds (PPTs) at baseline. (A) Association between pain intensity and non-evoked (spontaneous) pain duration in spinal cord injury (SCI); (B) association between pain intensity and number of neuropathic pain (NP) descriptors in SCI; (C) sex differences in PPTs over V2, C6, and L4 dermatomes according to noninjured (healthy control) and SCI groups. *N: Newtons. * p < 0.05; ** p < 0.01 (intragroup comparisons). Data are shown as mean and SE (standard error)*. Figure S2: Neuropathic descriptors evoked by the cold pressor test (12 °C 60 s, non-dominant hand or foot) or by the algometer (dominant hand or L4 dermatome) reported by participants with SCI with neuropathic pain (SCI-NP) and without neuropathic pain (SCI-noNP). (A) Neuropathic pain (NP) descriptors evoked by the cold pressor test (12 °C 60 s, non-dominant hand) in SCI-NP compared with SCI-noNP; (B) neuropathic pain (NP) descriptors evoked by the algometer (dominant hand) in SCI-NP compared with SCI-noNP; (C) neuropathic pain (NP) descriptors evoked by the cold pressor test (12 °C 60 s, non-dominant foot) in SCI-NP compared with SCI-noNP; (D) neuropathic pain (NP) descriptors evoked by the algometer (L4 dermatome, dominant side) in SCI-NP compared with SCI-noNP. *Data are presented as percentage for each group*. Table S1: Neuropathic descriptors evoked by the cold pressor test (12 °C 60 s, non-dominant hand) or by the algometer (dominant hand) reported by participants with SCI without neuropathic pain (n = 24); Table S2: Neuropathic descriptors evoked by the cold pressor test (12 °C 60 s, non-dominant hand) or by the algometer (dominant hand) reported by participants with SCI and neuropathic pain (n = 24); Table S3: Neuropathic descriptors evoked by the cold pressor test (12 °C 60 s, non-dominant foot) or by the algometer (L4 dermatome) reported by participants with SCI without neuropathic pain (n = 24); Table S4: Neuropathic descriptors evoked by the cold pressor test (12 °C 60 s, non-dominant foot) or by the algometer (L4 dermatome) reported by participants with SCI and neuropathic pain (n = 24).
