# Supplementary material for: Tonic Cold Pain Temporal Summation and Translesional Cold Pressor Test-Induced Pronociception in Spinal Cord Injury: Association with Spontaneous and Below-Level Neuropathic Pain
Source: Healthcare (Basel). 2024 Nov 17;12(22):2300. doi: 10.3390/healthcare12222300 (PMC11593809; doi:10.3390/healthcare12222300)
Supplement: Supplementary file 1 [file healthcare-12-02300-s001.zip › R¿¬os-Le¿«n_Supplementary Tables.pdf]

Supplemental Table S1. Neuropathic descriptors evoked by the cold pressor test (12ºC 60s, non-dominant hand) or by the algometer (dominant hand) reported by participants with SCI without neuropathic pain (n=24).

Subject number	AIS (A-D)	SCI neurological level	Mean Pain intensity during immersion (NRS: 0-10)	DN4 descriptors evoked by cold	Spasms painful sensation evoked by cold	DN4 descriptors evoked by PPT	Spasms painful sensation evoked by PPT
#1	A	T4	2.17	PC	Yes	No	No
#2	A	C5	2.5	PC	No	No	Yes
#3	A	T8	10	PC	No	No	No
#4	A	T4	7	PC	No	No	No
#5	A	T3	6.17	PC	No	No	Yes
#6	A	T6	2.29	Nb	No	No	No
#7	A	T4	10	PC	No	No	No
#8	A	T11	8.58	PC	No	No	No
#9	A	T10	1.17	Nb	No	No	No
#10	A	T7	0	Nb	No	T	No
#11	A	T5	1.67	T, P&N	No	No	No
#12	A	T4	0.67	Nb	No	No	No
n=12	12A	1C 11T	4.4±3.7	1 (1-1)	1Yes	0 (0-0)	2 Yes
#13	B	C8	9.17	PC, P&N	Yes	No	Yes
#14	B	C5	0	ES	Yes	ES	Yes
#15	C	T11	1.17	ES	Yes	ES	No
#16	C	T12	0	Nb	No	ES, I	No
#17	C	T11	1	Nb	No	No	Yes
#18	C	T5	7.75	PC	No	No	No
#19	C	T10	3.67	PC	No	B	No
#20	C	T9	1.17	PC	No	No	No
#21	D	T6	2.5	T, B	No	T	No
#22	D	T1	6.5	PC	No	No	Yes
#23	D	T9	4.17	Nb	No	PC	No
#24	D	T5	2.83	PC, Nb	No	No	No
n=12	2B 6C 4D	2C 10T	3.3±3	1 (1-1.25)	3 Yes	0.5 (0-1)	4 Yes
n=24	A-D	3C 21T	3.8±3.4	1 (1-1)	4 Yes	0 (0-1)	6 Yes

Data are presented as frequencies, mean ± SD, or median (25th and 75th percentiles). SCI: Spinal cord Injury; AIS: American Spinal Injury Association Impairment Scale; DN4: Douleur Neuropathique 4 Questions; PPT: pressure pain threshold, B: Burning, PC: Painful Cold, ES: Electric Shocks, T: Tingling, Pins and Needles: P&N, Nb: Numbness, I: Itching.

Supplemental Table S2. Neuropathic descriptors evoked by the cold pressor test (12ºC 60s, non-dominant hand) or by the algometer (dominant hand) reported by participants with SCI and neuropathic (n=24)

Subject number	AIS (A-D)	SCI neurological level	Mean Pain intensity during immersion (NRS: 0-10)	DN4 descriptors evoked by cold	Spasms painful sensation evoked by cold	DN4 descriptors evoked by PPT	Spasms painful sensation evoked by PPT
#25	A	T5	3.33	No	No	T	Yes
#26	A	T11	8.33	No	No	No	Yes
#27	A	T5	4.42	PC	No	No	Yes
#28	A	T4	2.5	T	No	No	No
#29	A	T1	4.5	No	No	No	Yes
#30	A	T4	0	No	No	No	No
#31	A	T5	1.5	No	No	I	Yes
#32	A	C7	5.25	PC	No	ES, T	No
#33	A	T10	9	No	No	No	No
#34	A	T8	6.2	PC	No	No	Yes
#35	A	T5	10	PC	No	P&N	No
#36	A	T4	3.5	B, PC, T, P&N, I, Nb	No	No	No
n=12	12 A	1C 11T	4.9±3	0.5 (0-1)	0 Yes	0 (0-1)	6 Yes
#37	B	C5	4.17	PC	Yes	No	No
#38	C	T10	9.08	No	No	No	No
#39	C	T11	7.42	No	No	No	No
#40	C	T1	7.75	No	No	ES	Yes
#41	C	T6	2.67	PC	No	No	No
#42	C	T4	10	PC	No	PC	No
#43	D	C5	1.83	T	Yes	T	Yes
#44	D	T10	4.33	No	No	I	Yes
#45	D	T8	8.33	No	No	ES	No
#46	D	T4	1.5	PC, Nb	No	No	No
#47	D	T4	0	ES, B	No	No	No
#48	D	T7	10	PC	No	P&N, ES	No
n=12	1B 4C 6D	2C 10T	5.6±3.6	1 (0-1)	2 Yes	0.5 (0-1)	3 Yes
n=24	24 A-D	2C 10T	5.2±3.3	1 (0-1)	2 Yes	0 (0-1)	9 Yes

Data are presented as frequencies, mean ± SD, or median (25th and 75th percentiles). SCI: Spinal cord Injury; AIS: American Spinal Injury Association Impairment Scale; DN4: Douleur Neuropathique 4 Questions; N: none; PPT: pressure pain threshold; B: Burning, PC: Painful Cold, ES: Electric Shocks, T: Tingling, Pins and Needles: P&N, Nb: Numbness, I: Itching.

Supplemental Table S3. Neuropathic descriptors evoked by the cold pressor test (12ºC 60s, non-dominant foot) or by the algometer (L4 dermatome) reported by participants with SCI without neuropathic pain (n=24).

Subject number	AIS (A-D)	SCI neurological level	Mean Pain intensity during immersion (NRS: 0-10)	DN4 descriptors evoked by cold	Spasms painful sensation evoked by cold	DN4 descriptors evoked by PPT (L4)	Spasms painful sensation evoked by PPT (L4)
#1	A	T4	0	No	Yes	No	No
#2	A	C5	0	No	No	No	Yes
#3	A	T8	3	T, PC	Yes	No	No
#4	A	T4	0	No	No	No	Yes
#5	A	T3	0	PC	No	No	Yes
#6	A	T6	0	No	No	No	No
#7	A	T4	0	No	Yes	No	No
#8	A	T11	0	No	No	ES	No
#9	A	T10	0	No	No	No	No
#10	A	T7	0	No	No	No	No
#11	A	T5	4	T, P&N	No	No	No
#12	A	T4	0	No	No	No	No
n=12	12A	1C 11T	0.6±1.4	0 (0-0.25)	3 Yes	0 (0-0)	3 Yes
#13	B	C8	1.3	No	Yes	No	Yes
#14	B	C5	3	No	No	ES	Yes
#15	C	T11	0	No	No	No	No
#16	C	T12	0	No	Yes	ES	No
#17	C	T11	0	No	Yes	No	Yes
#18	C	T5	9	PC	No	No	No
#19	C	T10	0	T	No	B	No
#20	C	T9	4	PC	No	No	No
#21	D	T6	0	No	No	B	No
#22	D	T1	3	PC	No	No	Yes
#23	D	T9	3	No	No	PC	No
#24	D	T5	9	No	Yes	No	No
n=12	2B 5C 4D	2C 10T	2.7±3.3	0 (0-1)	4 Yes	0 (0-1)	4 Yes
n=24	24 A-D	3C 21T	1.6 ±2.7	0 (0-1)	7 Yes	0 (0-0.25)	7 Yes

Data are presented as frequencies, mean ± SD, or median (25th and 75th percentiles). SCI: Spinal cord Injury; AIS: American Spinal Injury Association Impairment Scale; DN4: Douleur Neuropathique 4 Questions; N: none; PPT: pressure pain threshold; B: Burning, PC: Painful Cold, ES: Electric Shocks, T: Tingling, Pins and Needles: P&N, Nb: Numbness, I: Itching.

Supplemental Table S4. Neuropathic descriptors evoked by the cold pressor test (12ºC 60s, non-dominant foot) or by the algometer (L4 dermatome) reported by participants with SCI and neuropathic pain (n=24).

Subject number	AIS (A-D)	SCI neurological level	Mean Pain intensity during immersion (NRS: 0-10)	DN4 descriptors evoked by cold	Spasms painful sensation evoked by cold	DN4 descriptors evoked by PPT (L4)	Spasms painful sensation evoked by PPT (L4)
#25	A	T5	0	T	No	I	No
#26	A	T11	0	No	No	No	Yes
#27	A	T5	0	B, ES	No	ES	Yes
#28	A	T4	0	No	No	No	No
#29	A	T1	0	No	No	No	Yes
#30	A	T4	0.17	T	No	No	No
#31	A	T5	0	No	No	I	Yes
#32	A	C7	1	PC	No	ES, T	No
#33	A	T10	0.83	ES, T, Nb, B	No	T, Nb	No
#34	A	T8	4	P&N, I	No	No	No
#35	A	T5	2.33	No	No	P&N	No
#36	A	T4	1.5	B, P&N, T, Nb	No	No	No
n=12	12A	1C 11T	0.8±1.2	1 (0-2)#	0 Yes	0.5 (0-1)	4 Yes
#37	B	C5	3.5	T, P&N, ES	No	T	No
#38	C	T10	0	No	No	No	No
#39	C	T11	2.33	Nb	No	No	No
#40	C	T1	3.33	B, Nb	No	No	Yes
#41	C	T6	0	No	Yes	No	Yes
#42	C	T4	4.83	PC	No	No	No
#43	D	C5	6.25	PC	No	T	No
#44	D	T10	4.17	T, Nb	No	I	Yes
#45	D	T8	8.83	PC	No	ES	Yes
#46	D	T4	1.5	PC	No	No	No
#47	D	T4	0	ES, T	No	No	No
#48	D	T7	1.17	No	No	No	No
n=12	1B 4C 6D	2C 10T	3.0±2.7	1 (0.75-2.0)*	1 Yes	0 (0-1)	4 Yes
n=24	24A.D	3C 21T	1.9±2.4	1 (0-2)**	1 Yes	0 (0-1)	8 Yes

Data are presented as frequencies, mean ± SD, or median (25th and 75th percentiles). SCI: Spinal cord Injury; AIS: American Spinal Injury Association Impairment Scale; DN4: Douleur Neuropathique 4 Questions; N: none; PPT: pressure pain threshold; B: Burning, PC: Painful Cold, ES: Electric Shocks, T: Tingling, Pins and Needles: P&N, Nb: Numbness, I: Itching. #-p=0.08; *p<0.05; **p<0.001 (Mann-Whitney U test: comparison with data presented in Table 5).
