# Supplementary material for: Tonic Cold Pain Temporal Summation and Translesional Cold Pressor Test-Induced Pronociception in Spinal Cord Injury: Association with Spontaneous and Below-Level Neuropathic Pain
Source: Healthcare (Basel). 2024 Nov 17;12(22):2300. doi: 10.3390/healthcare12222300 (PMC11593809; doi:10.3390/healthcare12222300)

A. NP descriptors evoked by the cold pressor test (60s 12°C, non-dominant hand)

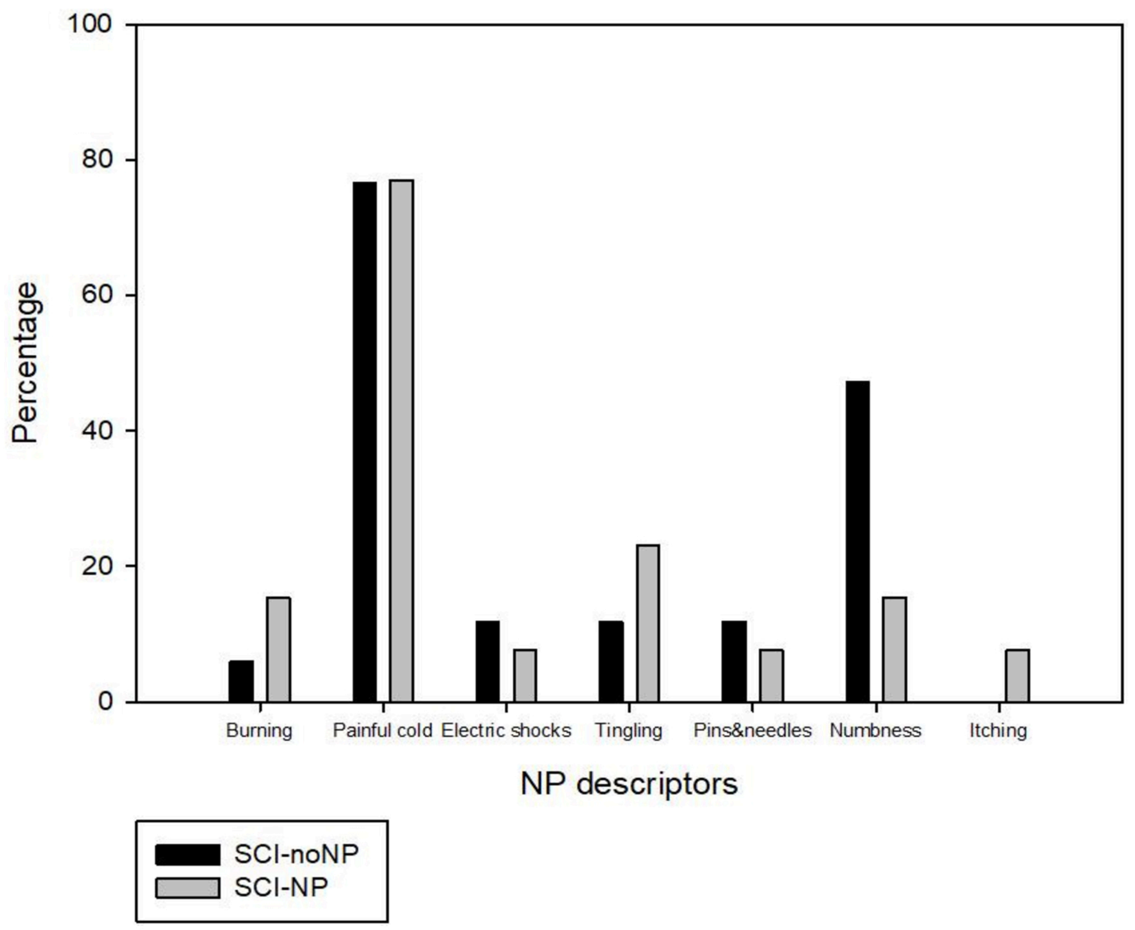

B. NP descriptors evoked by the algometer (dominant hand)

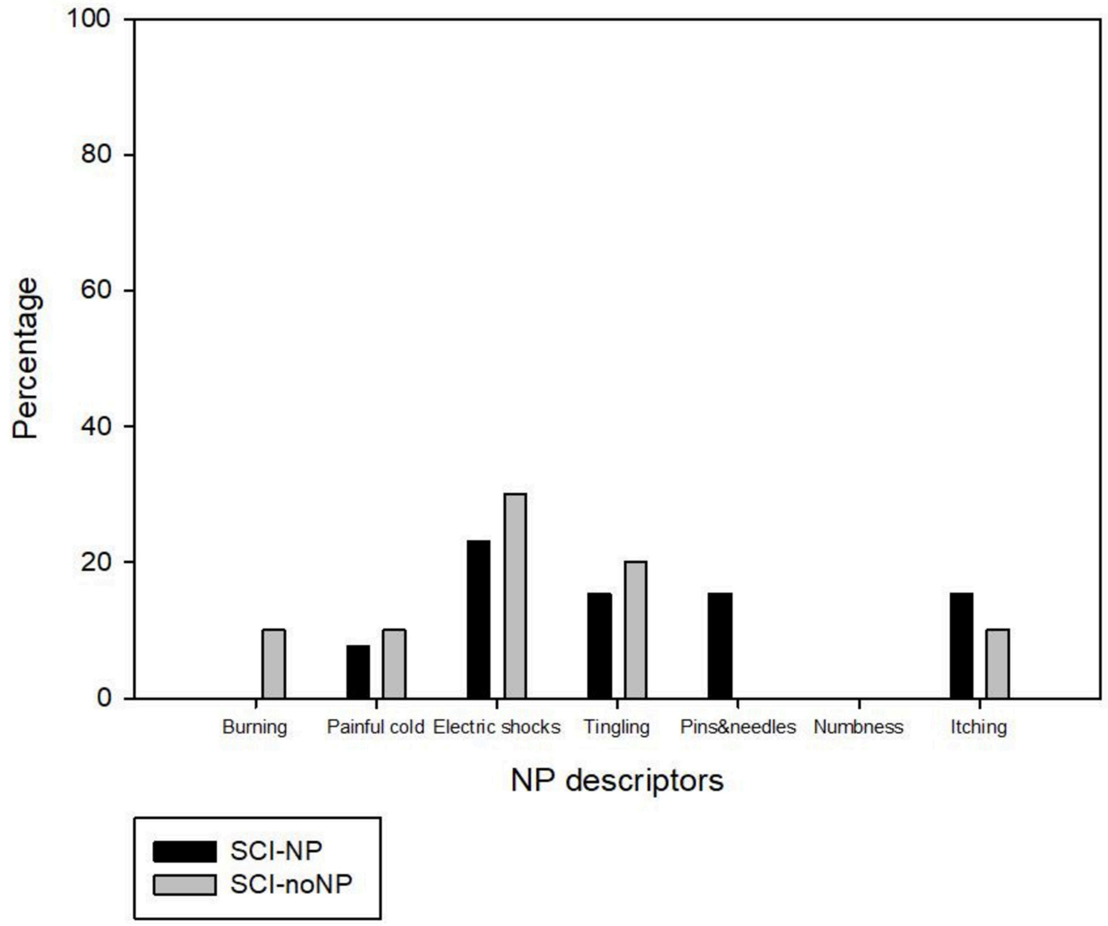

C. NP descriptors evoked by the cold pressor test (60s 12°C, non-dominant foot)

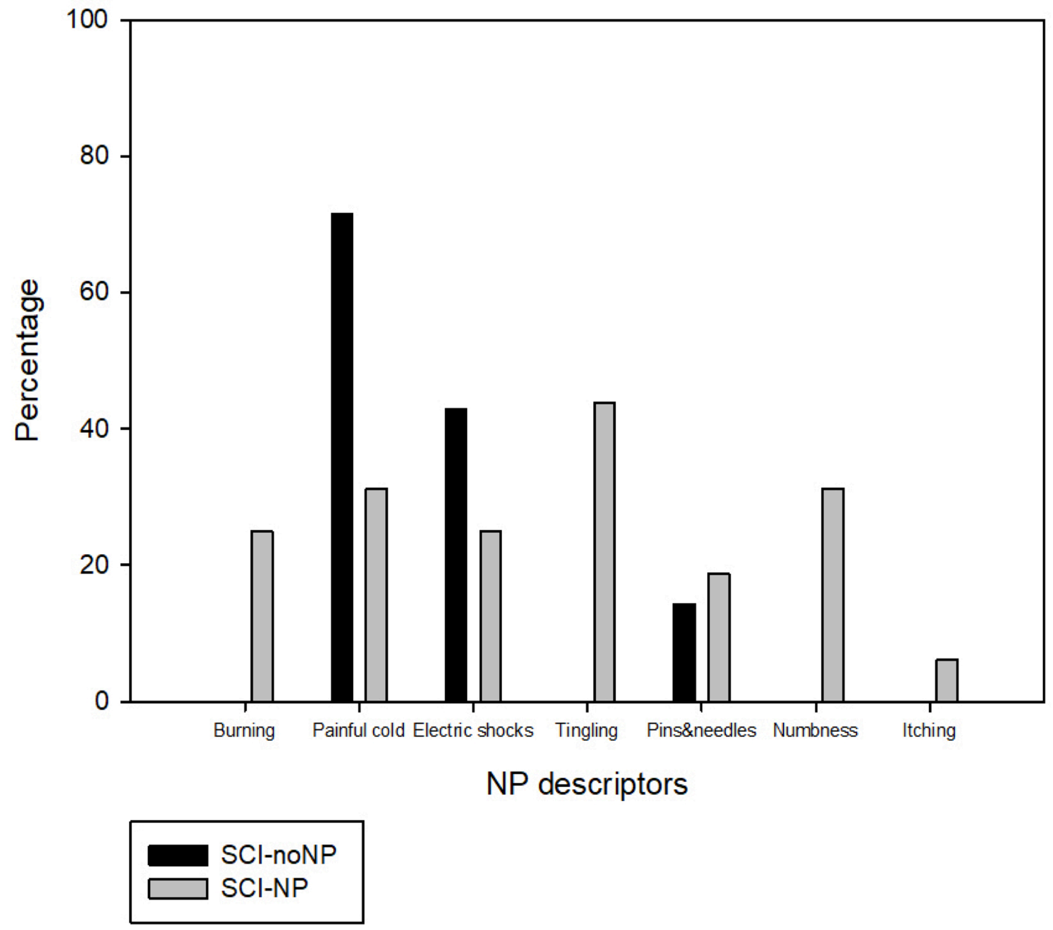

D. NP descriptors evoked by the algometer (L4 dermatome)

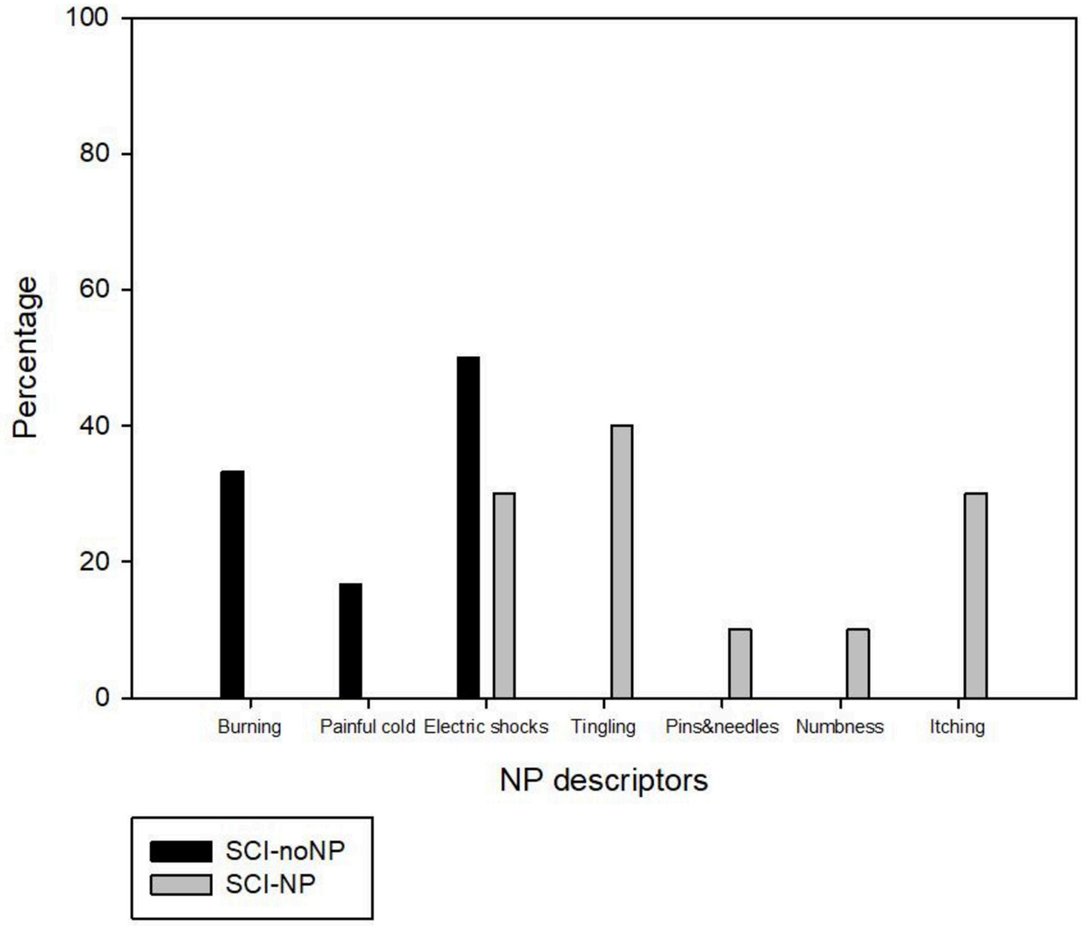

Supplement: Supplementary file 1 [file healthcare-12-02300-s001.zip › Supplementary Figure 2.pdf]
