# Supplementary figures and images for: Tonic Cold Pain Temporal Summation and Translesional Cold Pressor Test-Induced Pronociception in Spinal Cord Injury: Association with Spontaneous and Below-Level Neuropathic Pain
Source: Healthcare (Basel). 2024 Nov 17;12(22):2300. doi: 10.3390/healthcare12222300 (PMC11593809; doi:10.3390/healthcare12222300)

**A**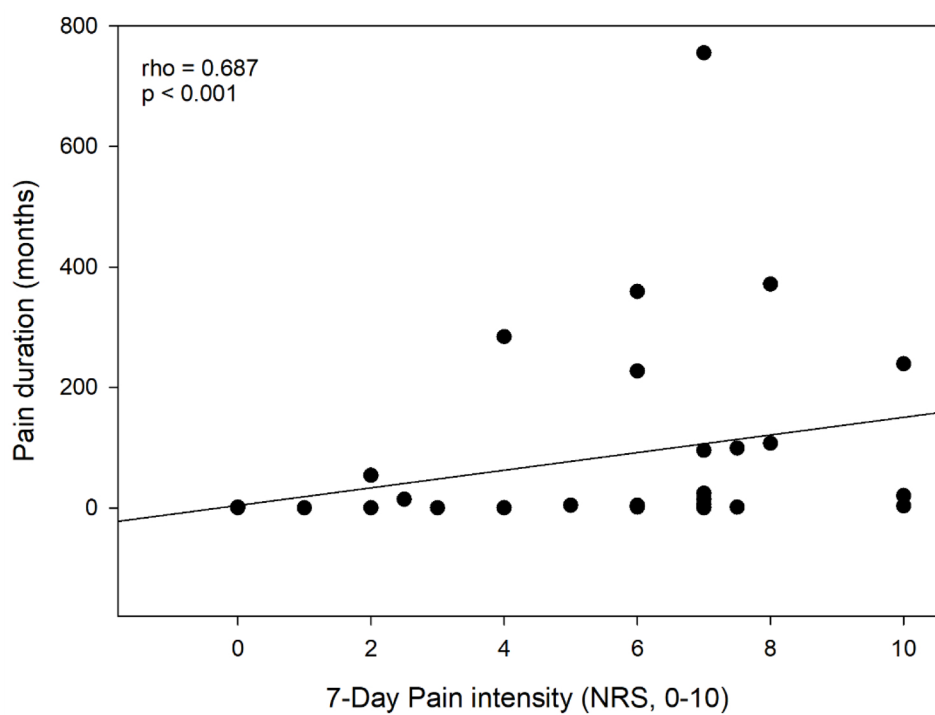**B**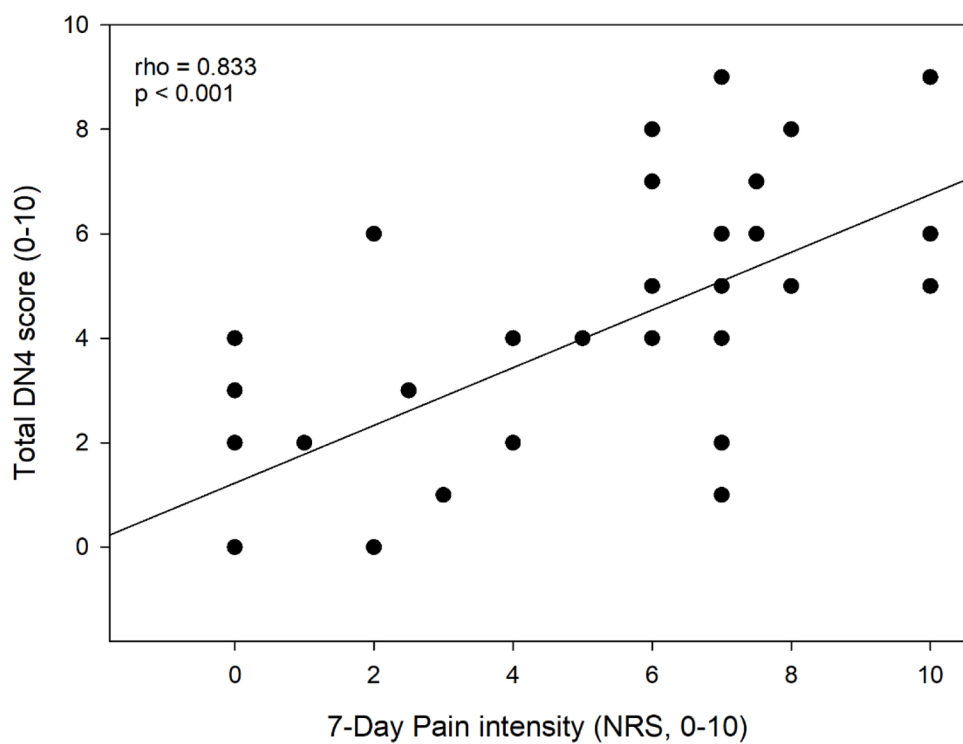**C**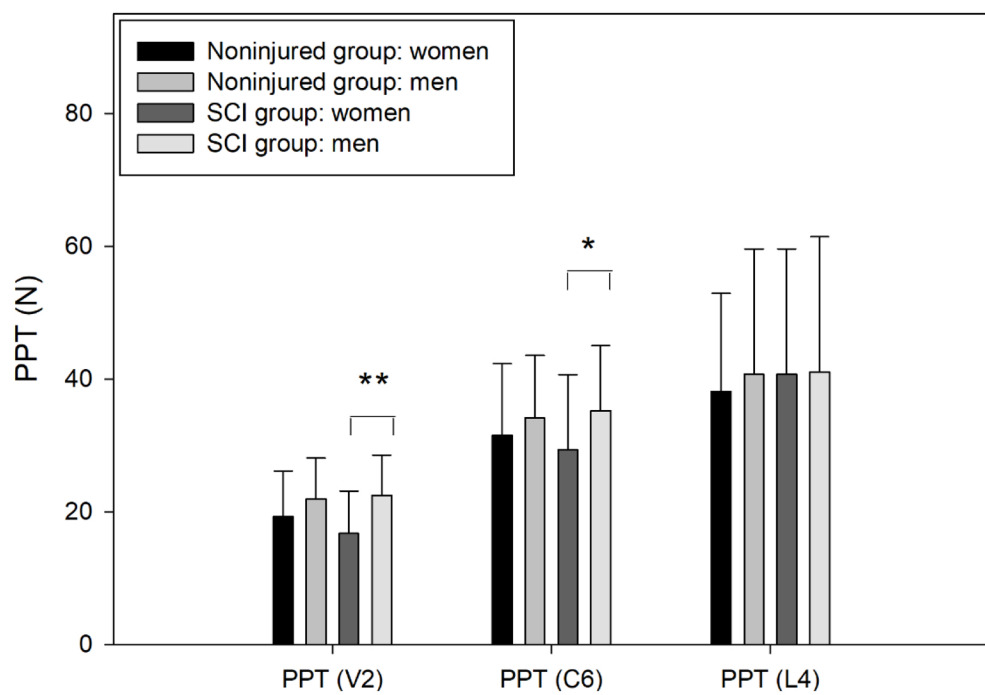

Supplement: Supplementary file 1 [file healthcare-12-02300-s001.zip › Supplementary Figure 1.pdf]
